# A review of wide surgical excision of hidradenitis suppurativa

**DOI:** 10.1186/1471-5945-12-9

**Published:** 2012-06-26

**Authors:** Ziyad Alharbi, Jens Kauczok, Norbert Pallua

**Affiliations:** 1 Department of Plastic and Hand Surgery-, Burn Unit, Medical Faculty, RWTH Aachen University, Aachen, Germany

**Keywords:** Hidradenitis suppurativa, Acne inversa, Follicular diseases, Surgical reconstruction, Wide surgical excision

## Abstract

**Background:**

Hidradenitis suppurativa (HS) is a chronic inflammatory cutaneous disorder that involves the infundibular terminal follicles in areas rich of apocrine glands. It can be associated with fistulating sinus, scarring and abscesses formation. Hidradenitis suppurativa is a challenging aspect and requires a proper treatment plan which may involve different specialties. We present herein the option of surgical treatment involving wide surgical excision and methods of reconstruction as well as the rate of recurrence. Furthermore, review of the literature regarding surgical treatment of hidradenitis suppurativa is provided.

**Methods:**

A retrospective analysis reviewed 50 operative procedures for 32 patients in 5 anatomical sites. These anatomical sites have been divided to 23 sites involving the axilla, 17 sites involving the inguinal region and 8 sites involving the perianal/perineal area, 1 site involving the gluteal region and 1 site involving the trunk region.

**Results:**

Twenty six patients (81, 25 %) showed no recurrence after surgery and the average time of hospital stay period was 5 days. Recurrence was observed only in 6 patients (18, 75 %).

**Conclusion:**

Elimination of the acute inflammatory process should occur in advance, including the use of antibiotics and minor surgeries such as abscess drainage with proper irrigations. After stabilizing the acute phase, wide surgical excision is recommended. Herein, planning of surgical reconstruction should be initiated to achieve the best outcome and consequently decreasing the risk of recurrence and complications after surgery.

## Background

Hidradenitis suppurativa (HS) is a chronic inflammatory cutaneous disorder that involves the infundibular terminal follicles in areas rich of apocrine glands and associated with formation of abscesses and fistulating sinus [1-5]. The pathogenesis of the disease is not fully understood, although it was reported that HS is androgen dependent that can be associated with endocrine abnormalities [6]. Bacterial infection is considered as a secondary event in the pathogenesis. Furthermore, smoking and obesity are both known as risk factors and may increase the severity of the disease [1,6].

Clinical manifestations include painful nodules, abscesses, sinus tracts, and ropelike hypertrophic scars in the apocrine gland-bearing areas [7]. Consequently, the abscesses extend deeper into the subcutaneous tissue and then intercommunicating sinus tracts develop, resulting in irregular hypertrophic scars [8]. Hidradenitis suppurativa was initially classified by using Hurley's Staging System (Table 1) [9].

**Table 1:** **Hurley’s Staging System**[10]

**Stage**	**Characteristics**
I	Solitary or multiple isolated abscess formation without scarring or sinus tracts.
II	Recurrent abscesses, single or multiple widely separated lesions, with sinus tract formation. (Require minor surgery such as incision and drainage.)
III	Diffuse or broad involvement across a regional area with multiple interconnected sinus tracts and abscesses

Likewise, Sartorius et al. have suggested that the Hurley system is not enough to assess the efficacy of the treatment. Therefore, they described the Sartorius Staging System. Points are accumulated in each category to assess the treatment of HS in an accurate way [11]. The Sartorius Staging System [12] accumulates points according to:

· Anatomic regions involved

· Number and types of lesions involved (abscesses, nodules, fistulas, scars, points for lesions of all regions involved)

· The distance between lesions, in particular the longest distance between two relevant lesions (i.e. nodules and fistulas in each region or size if only one lesion present)

· The presence of normal skin in between lesions

It is indeed a challenging aspect and requires a proper treatment plan that may involve different specialties. Hidradenitis suppurativa is commonly misdiagnosed and sometimes even referred to many subspecialties [13]. In general, treatment includes the use of topical or systemic antibiotics, topical antiseptics and intralesional corticosteroids. Furthermore, systemic retinoids, antiandrogen therapy, immunotherapy (TNF alfa inhibitors) and oral immunosuppressive agents have also shown a positive effect on disease progression [10,12]. However, for most cases of advanced hidradenitis suppurativa, radical surgery can be the only curative treatment option [14]. It is also reported that early wide surgical excision is important and effective in order to prevent complications and the recurrence of hidradenitis suppurativa and to improve the quality of life [15].

This article focuses on the surgical treatment of hidradenitis suppurativa with special regard to the methods of reconstruction for resulted defects after wide surgical excision in the axilla, inguinal region, gluteal region, trunk, perineal and perianal area. The rate of recurrence will be also reviewed. Furthermore, a review of the literature regarding surgical treatment of hidradenitis suppurativa is provided.

## Methods

This retrospective analysis reviewed 32 patients with chronic inflammatory moderate to severe hidradenitis suppurativa (Hurley grade II and III) treated in our hospital from 2003 to 2009 (Table 2). Follow up of all patients has been conducted in our out patient department (OPD) with a mean period of 24 months after surgery. 50 operative procedures were retrospectively reviewed in 5 anatomical sites, 23 sites involving the axilla, 17 sites involving the inguinal region and 8 sites involving the Perianal/perineal area, 1 site involving the gluteal region and 1 site involving the trunk region. The regional ethics committee advised that approval was not necessary for this retrospective analysis. However, all patients have provided written informed consent for the publication of their clinical details and any accompanying clinical images.

**Table 2:** shows the recurrence rate with the type of surgical reconstruction in each site

Location of Defects (total number:50)	Methods of reconstruction	Percentage of usage related to each region	Recurrence
Axilla	Rotation fasciocutaneous flap	30,4 %	39,6 %
		(7 sites)	(2 patients)
46 %	Transposition fasciocutaneous flap (Limberg flap)	39,1 %	11,1 %
	Parascapular fasciocutaneous flap	(9 sites)	(1 patient)
		21,7 %	0 %
	TDAP flap	(5 sites)	
	ALTP flap	4,3 %	0 %
		(1 site)	
		4,3 %	0 %
		(1 site)	
Inguinal	Primary closure	47 %	12,5 %
34 %	Split-thickness skin graft	(8 sites)	(1 patient)
	Rotation fasciocutaneous flap	17,75 %	33,3 %
	Transposition fasciocutaneous flap	(3 sites)	(1 patient)
		17,75 %	33,3 %
	Abdominoplasty	(3 sites)	(1 patient)
		11,8 %	0 %
		(2 sites)	
		5,8 %	0 %
		(1 site)	
Perianal/ Perineal 16 %	Transposition fasciocutaneous flap	75 %	0 %
	Gracilis musculocutaneous flap	(6 sites)	
		25 %	0 %
		(2 sites)	
Gluteal	Split-thickness skin graft	100 %	0 %
2 %		(1 site)	
Trunk	Split-thickness skin graft	100 %	0 %
2 %		(1 site)	

Twelve patients (37,5 %) were males and 20 patients (62,5 %) were females. The age at the time of presentation ranged from 17 years to 51 years (Table 3). Overall, 22 patients (68,75 %) were obese. From this category, 5 patients (15,6 %) had Body Mass Index (BMI) of more than 25 and 14 patients (43,75 %) had BMI of more than 30. Two patients (6,2 %) represented BMI of more than 35 and only one patient (3,1 %) represented BMI of more than 40. The analysis also showed that 10 patients (31,25 %) were smokers (5 males and 5 females).

**Table 3:** Patients characteristics with Age, Body Mass Index, Smoking and Size of Defect

Pat. No.	Sex	Age	BMI	Smoking	Site	Defect Size (cm)	Recurrence
1	F	46	33.6	No	Axilla	11 × 5,2 × 2	-
2	M	35	31.2	Yes	Axilla	14 × 6,2 × 4	-
3	M	32	30.1	Yes	Inguinal/Perianal	10,5 × 8 × 3,1	-
4	F	30	24.6	No	Axilla	7× 4 × 2,5	-
5	F	32	26.3	No	Inguinal/Perineal	10 × 4.5 × 2,3	-
6	F	39	29.8	No	Axilla	6.3 × 5.8 ×1,5	-
7	F	21	24.9	No	Axilla	6 × 5 × 1,5	-
8	M	36	33.2	Yes	Inguinal	8 × 3 × 1	+−
9	F	24	33.7	No	Axilla/Trunk	11 × 4 × 1,5	-
10	F	31	20.4	No	Inguinal	9 × 3 × 1,5	+−
11	F	38	30.1	Yes	Bilateral Axilla	6,8 × 6 × 2,5	+−
						7 × 5,8 × 2,3	
12	M	29	24.0	Yes	Bilateral Axilla	3,5 × 4 × 1	+−
						4 × 4 × 0,5	
3	M	15	32.4	No	Bilateral Axilla	7 × 6 x 28 × 5 ×1,5	-
14	M	21	24.8	No	Axilla	12 × 4 × 0,8	-
15	F	37	38.8	No	Bilateral Axilla	8 × 6 × 2,3	-
						7 × 5 × 2,5	
16	F	26	41.2	No	Axilla	10 × 8 × 2,5	-
17	M	41	32.4	Yes	Inguinal	5,5 × 3× 2	-
18	M	34	21.9	Yes	Inguinal	8 × 4 × 3,5	+
19	F	25	31.2	No	Inguinal	5 × 2 × 1	-
20	F	51	28.7	Yes	Perineal/Perianal	8 × 4 × 2	-
21	M	26	27.8	Yes	Axilla	9 × 7 ×3	+−
22	F	43	31.6	No	Axilla	8,6 × 5 × 1,5	-
23	F	36	34.0	No	Bilateral Perianal/Perineal	20 × 13 × 5	--
						8 × 6 × 3	
24	F	22	20.4	Yes	Bilateral Inguinal	11,5×2,5×1,5	-
						2,2×,8×,5	
25	F	40	24.0	Yes	Inguinal	6,5 × 4 × 0,8	-
26	F	30	18.4	No	Inguinal	7 × 4,5 × 0,5	-
27	M	20	26.0	No	Axilla	9 × 7 × 3	-
28	F	28	22.9	No	Inguinal	3,7 × 1 × 2,1	-
29	M	50	31.1	Yes	Bilateral Axilla	5,6×5,1,1,2	--
						2,6 × 0,5 ×0,5	
30	F	22	30.1	No	Bilateral Inguinal	14 × 3 × 1,5	-
						15 × 2 × 1,5	-
31	F	25	30.6	No	Inguinal und Mons pubis	17 × 5 × 2,5	-
						13 × 4 × 1,5	
32	M	21	22.4	Yes	Axilla	13 × 5 × 1,5	-

The patients had chronic inflammatory hidradenitis suppurativa (Hurley grade II and III). Intravenous antibiotics were prescribed depending on wound tissue swab and laboratory results. Minor surgeries such as abscess drainage and irrigation were performed in order to irrigate the wounds. Further irrigation has been performed in the surgical ward prior to the surgical procedure. Wide surgical excisions were carried out for all patients with wide margin (i.e. 1 cm) and deep margin including the skin, subcutaneous tissue till reaching fascia. Stool Management System (SMS) has been used to prevent contamination of the stool to the surrounded skin otherwise colostomy was conducted for selected patients with perianal or perineal lesions. Then, reconstruction techniques were conducted based on the site and the size of the defects including primary closure, skin grafting, local flaps as random pattern or pedicle pattern, regional flaps and the more invasive free (microvascular) tissue transfer in selected patients. The size of defects resulted after excision ranged from 16,5 cm2 (5,5 x 3 cm) to 735 cm2 (35 x 21 cm). At the time of surgery, all patients have been administrated with single dose intravenous antibiotics depending on the previous swab results. One-stage operations: wide surgical excision and reconstruction of defect was performed for 26 patients (81,25 %). Two-stages operations have been performed for 5 patients (15,63 %) buy using the negative pressure therapy in one stage and the reconstructive art in the other stage. Only one patient has been operated in three stages (3,12 %) which included the use of negative pressure therapy in one stage and other debridement with irrigation due to persistent pus secretions and then the reconstruction of defect in the third stage.

For the axilla, rotation fasciocutaneous flap was performed in 7 sites (30,4 %), and transposition fasciocutaneous flap including the Limberg flap was used in 9 sites (39,1 %). Parascapular fasciocutaneous flap was selected in 5 patients (21,7 %) and the thoracodorsal artery perforator (TDAP) flap was the option in 1 patient (4,3 %). Anterolateral thigh perforator (ALTP) flap has been used in 1 patient (4,3 %).

For inguinal lesions, primary closure was used in 8 sites (47 %) and 3 Split-thickness skin grafting techniques (17,75 %) were performed. Rotation fasciocutaneous flap was used in 3 sites (17,75 %) and transposition fasciocutaneous flap was carried out in 2 sites (11,8 %). Only one patient (5,8 %) was treated with abdominoplasty for reconstructing the defect involving the both inguinal areas and Mons pubis.

Reconstruction of the perianal and the perineal regions included the using of transposition fasciocutaneous flap in 6 sites (75 %) and Gracilis musculocutaneous flap was used in 2 patients (25 %). Reconstruction of the trunk and gluteal region was carried out for only two patients by performing a split thickness skin graft for each site.

## Results

Daily dressing has been performed in a sterile concept. Twenty eight Patients (87,5 %) showed no complications after surgery. The average time of hospital stay period was 5 days. Physiotherapy and post-operative rehabilitation were also started.

After follow up (mean follow up time is 24 months), 26 Patients (81,25 %) showed no recurrence. Recurrence rate (Tables 2 and 3) was observed in 6 patients (18,75 %). The recurrence rate was seen in patients with Hurley grade III and 5 smokers out of 6 patients in this category (83,33 %).

**Figure 1:**
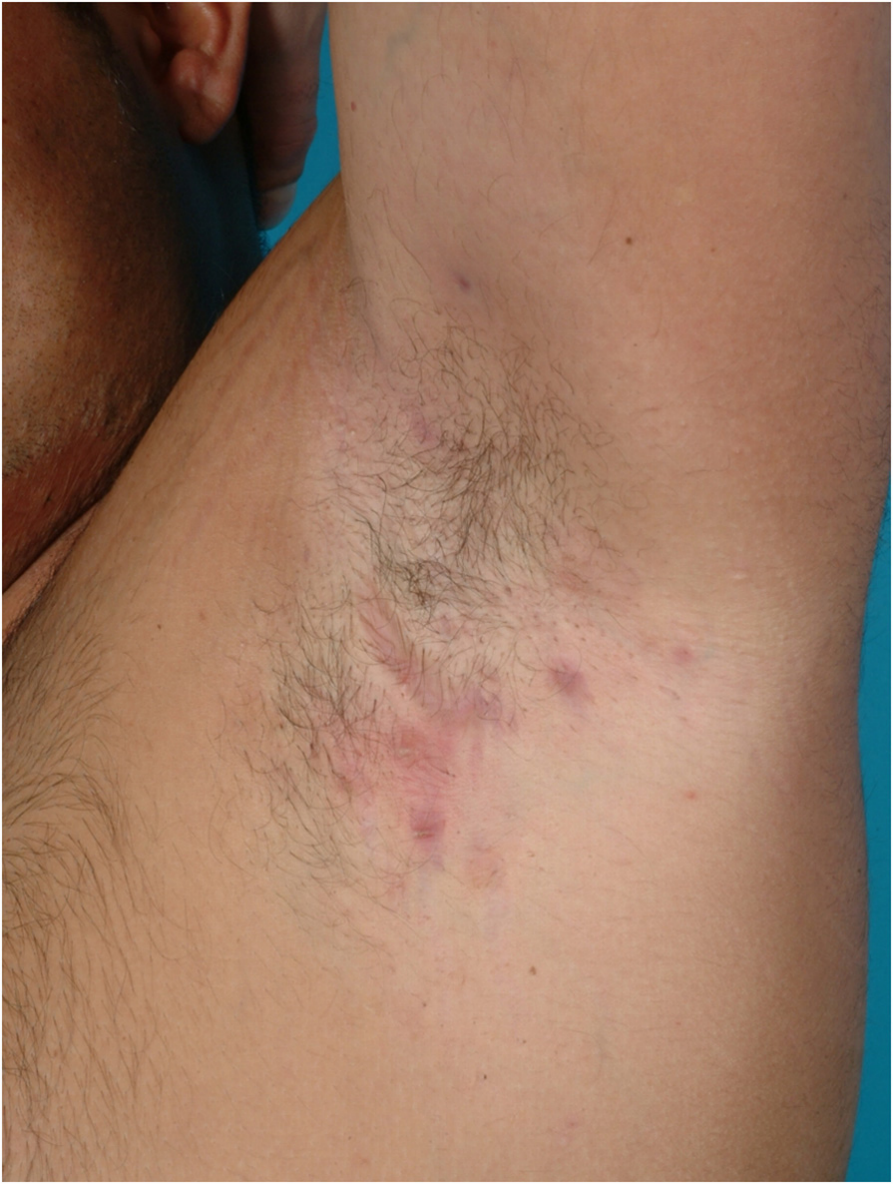
Hidradenitis suppurativa (Hurley's Staging II) in the left axilla.

**Figure 2:**
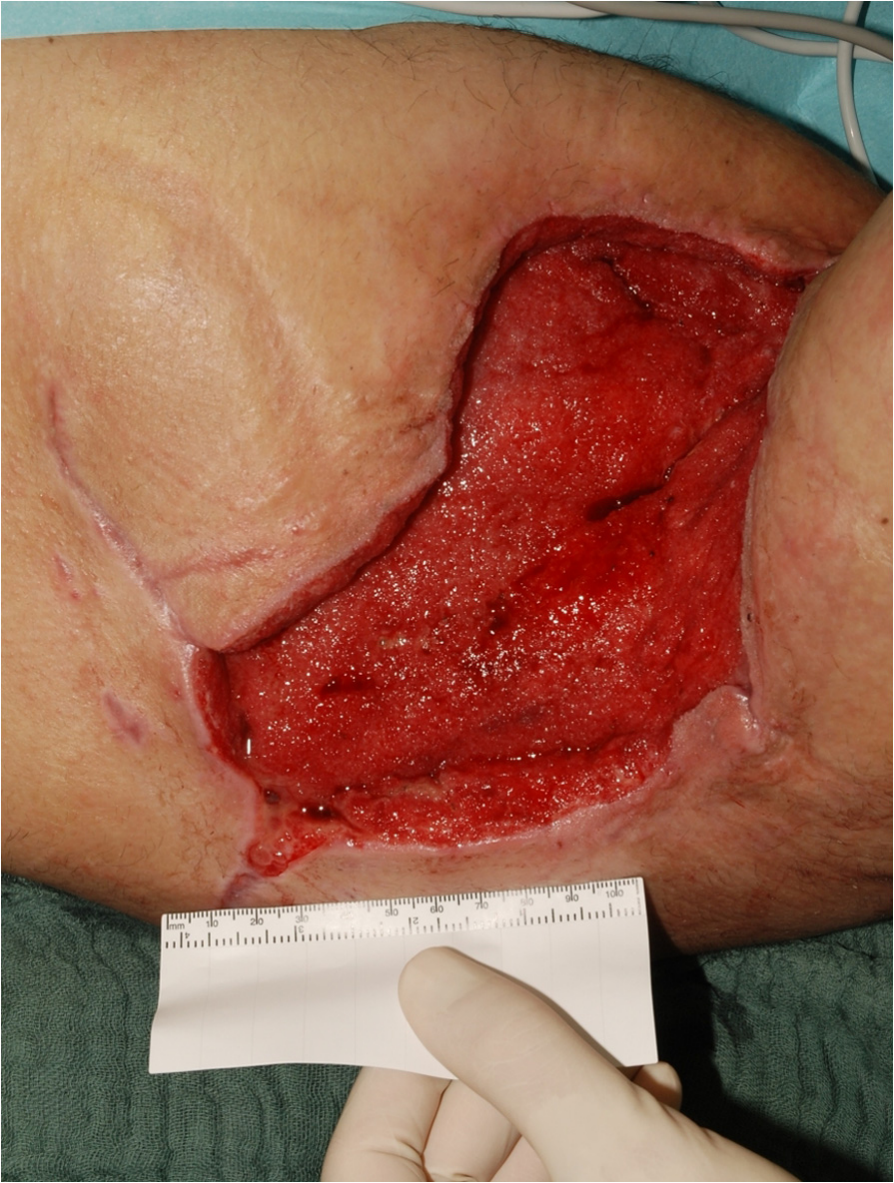
The resulted defect after wide surgical excision.

**Figure 3:**
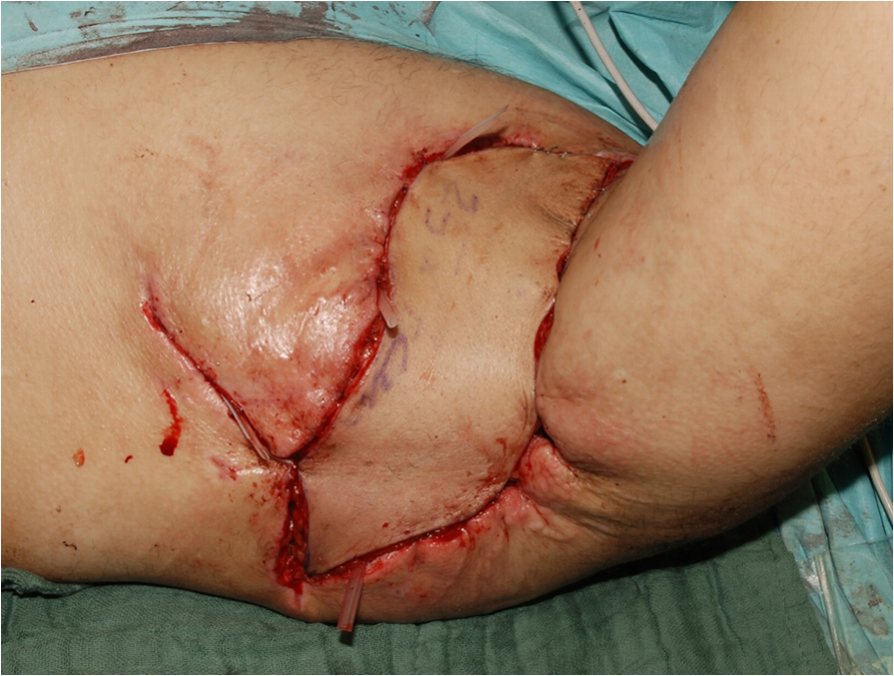
Adaptation of the ALTP flap after vascular anastomosis.

**Figure 4:**
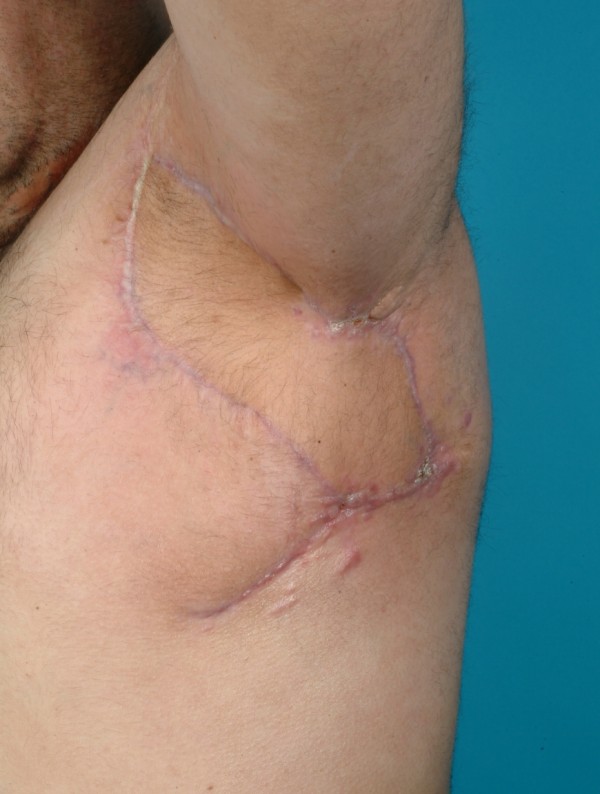
Post-operative photo demonstrates no healing abnormalities.

Reconstruction of the axillary region included several techniques but primary closure and skin grafting were not performed based on expected complications such as contractures, excessive scarring. For those lesions in this area, rotation fasciocutaneous flap and transposition fasciocutaneous flap such as Limberg flap were performed successfully and showed good aesthetic as well as function results. Parascapular fasciocutaneous flap and even thoracodorsal artery perforator (TDAP) flap were selected for moderate to diffuse lesions. Massive lesions in the axillary region were reconstructed in 1 of our patients by the use of free (microvascular) tissue transfer in the form of anterolateral thigh perforator (ALTP) flap and showed good functional as well as aesthetic results (Figures 1, 2, 3, and 4).

Reconstruction of the inguinal region included primary closure for mild and moderate lesions. Likewise, split-thickness skin grafting was selected for large defects. Rotation fasciocutaneous flap and transposition fasciocutaneous flap were performed successfully and showed good aesthetic results. For bilateral inguinal lesions and involvement of Mons pubis, an abdominoplasty was performed for one patient and showed good outcome.

**Figure 5:**
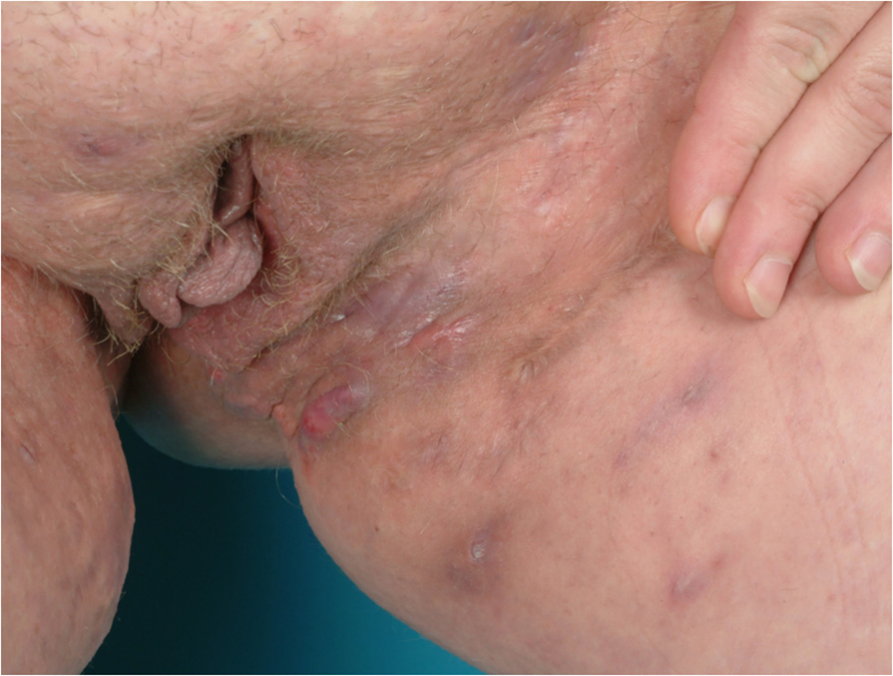
Bilateral Hidradenitis suppurativa (Hurley's Staging III) in the perianal, perineal and mons pubis showing the left side.

**Figure 6:**
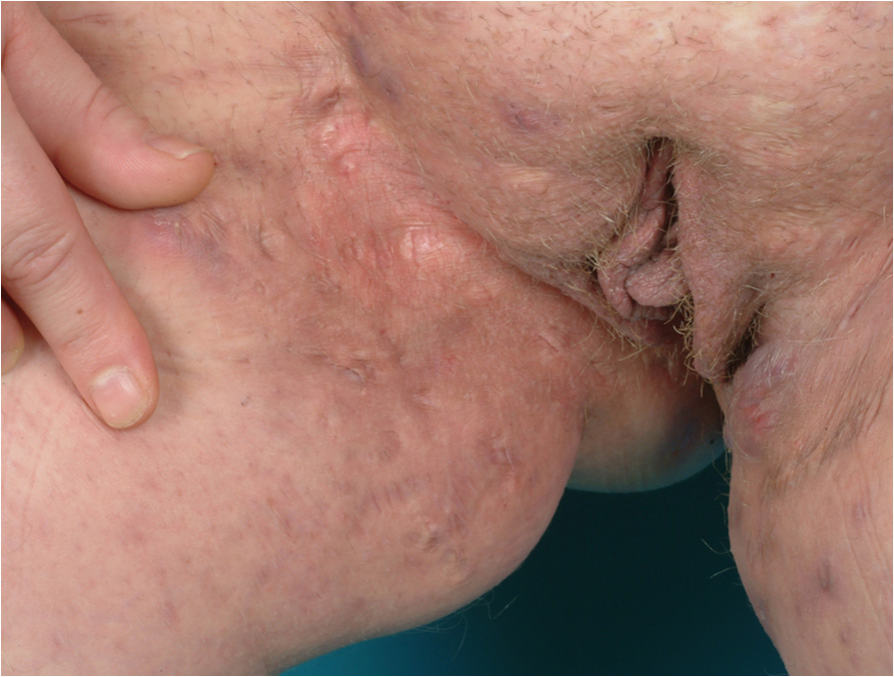
Bilateral Hidradenitis suppurativa (Hurley's Staging III) in the perianal, perineal and mons pubis showing the right side.

**Figure 7:**
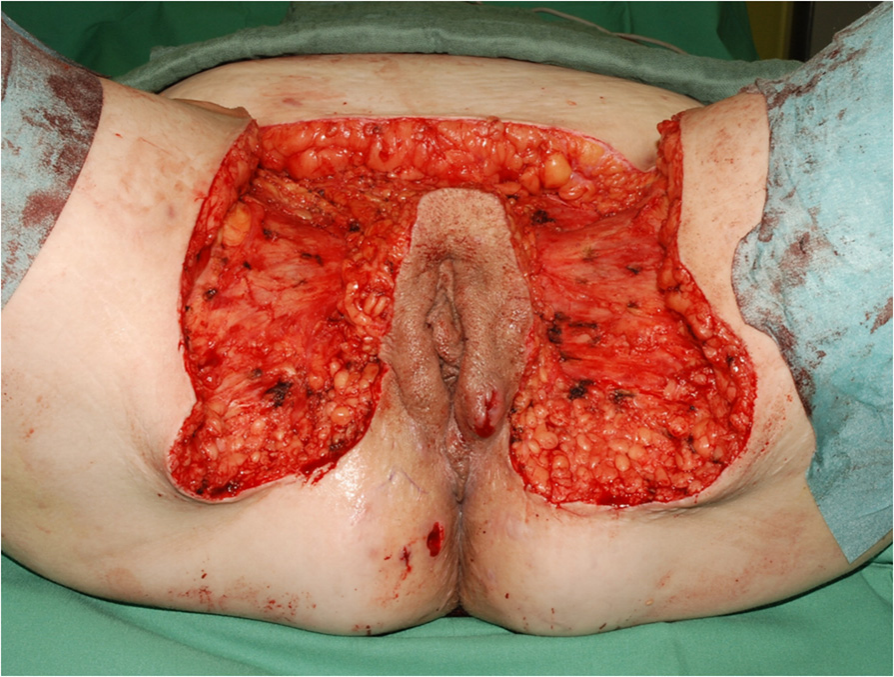
The resulted defect after wide surgical excision.

**Figure 8:**
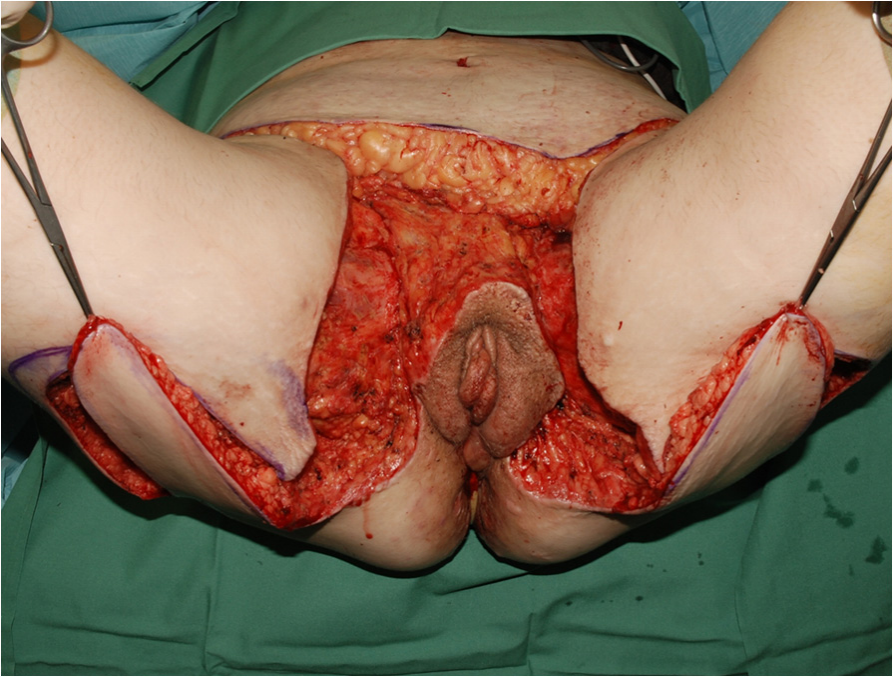
Bilateral transposition flap used to cover large defects bilaterally.

**Figure 9:**
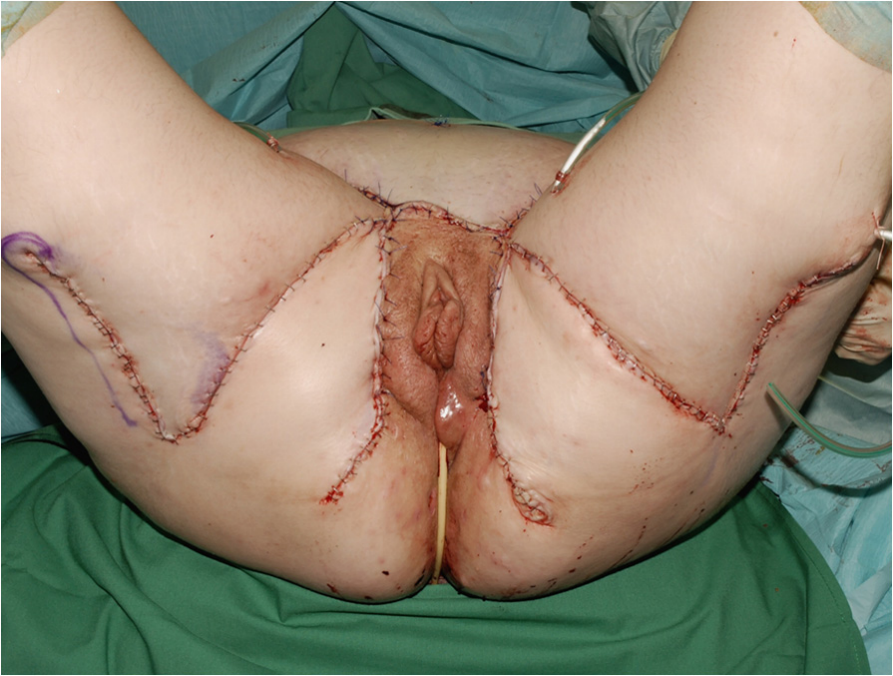
The post-operative result of the bilateral trasposition flap.

For lesions located on the perianal and the perineal regions, several techniques have been carried out including the use of bilateral transposition flap (Figures 5, 6, 7, 8, and 9) or the using of Gracilis musculocutaneous flap for severe lesions. Both techniques required preservation of the vulva and the anal sphincter. For lesions located in the gluteal and the trunk area, skin grafting was used successfully.

## Discussion

The literature on surgical treatment of hidradenitis suppurativa is huge and the review of this disease goes back to the 1950s [16-19]. Recently, many articles have been published regarding this point and some recent key references exist regarding surgical treatment of hidradenitis suppurativa [20,21]. Primary closure, healing by secondary intention and skin grafting are considered to be the most widely used procedures. Furthermore, several surgical techniques depending on secondary intention for minor or extensive disease are also described in the literature [21].

In fact, 18,75 % recurrence rate can be considered high after extensive surgery procedures. On the other hand, this recurrence rate was strongly associated with the extent (Hurley grade) of disease. Herein, in very advanced cases with Hurley grade III, it can be very hard to radically remove all HS tissue even if very extensive surgery is applied and at least minor recurrence is expected and accepted. Sartorius score cannot, but Hurley classification with clinical margin evaluation can possibly give valuable information for proper treatment options.

Excision and split skin grafting is a basic tool in the surgical treatment and the result of this procedure is often satisfactory [22-24]. Massive regional hidradenitis suppurativa can be successfully managed with wide surgical excision, VAC therapy, and skin grafting for better outcomes [25]. Furthermore, Negative-pressure dressings have been used as bolster for skin grafts in order to reconstruct such defects after wide surgical excision [26,27].

However, the use of flaps to prevent less favorable functional results was introduced at an early stage. A review of the Limberg flap for axillary hidradenitis was presented quite recently [28]. Local fasciocutaneous V-Y advancement flaps was reported for large defects following wide surgical excision of long-standing hidradenitis suppurativa of the axilla [29]. Other option is the double opposing V-Y perforator-based flaps which have been described for reconstruction of axillary defects following excision of hidradenitis suppurativa to recreate the axillary contour after wide surgical excision of the hair-bearing skin of the axilla [30]. More options exist like the use of a versatile transpositional flap for axillary hidradenitis suppurativa [31]. Some flaps may be indicated in particular cases such as the use of thoracodorsal artery perforator flap (TDAP) in axillary hidradentitis suppurativa [32,33]. Herein, lateral thoracic fasciocutaneous island flap was also used for treatment of recurrent hidradenitis axillaris suppurativa and other axillary skin defects [34].

The pedicled gracilis myocutaneous flap has been introduced as a surgical treatment of hidradenitis suppurativa of the groin and perineum [35]. It was even proposed that the medial thigh lift to be considered for immediate defect closure after radical excision of localised inguinal hidradenitis suppurativa provided that no perifocal signs of infection are present after debridement [36]. Furthermore, modified abdominoplasty was also reported as a functional reconstruction for recurrent hidradenitis suppurativa of the lower abdomen and groin [37]. The anterolateral thigh (ALT) flap has been reported for reconstruction of groin and vulval hidradenitis suppurativa [38]. Furthermore, the anterior Obturator Artery Perforator (aOAP) flap seems to be a save option for the reconstruction of perineal defects after wide surgical excision of hidradenitis suppurativa [39], although it was not introduced specifically for this disease.

It should be noted that the use of colostomy is not an absolute indication for treating such defects in the perianal or perineal region. We believe that flaps in these areas are more susceptible to infections. Colostomy can be performed but should be preserved for selected patients with massive extensive defects. Some patients do not agree with colostomy and, thus the consent of this procedure does not apply in many cases. However, this does not interfere with the selected treatment plan.

For buttocks, more options have been stated in the literature such as the fasciocutaneous flaps in gluteal hidradenitis suppurativa [40]. Other options were also documented such as the extended split superior gluteus maximus musculocutaneous flap. This flap is easy to harvest and leaves aesthetically satisfactory results [41].

There is no doubt that this approach of treatment is mainly dependent on the size and the site of the defect. Despite the method of reconstruction, the hospitalization period can be reduced and, thus reducing the cost of treatment. This goal can be elusive and therefore radical excision and more advanced reconstruction techniques are performed in order to close defects in a permanent way. We found that wide surgical excision as well the direct closure technique showed better outcome and limited the cost of treatment and the hospitalization period as well as the recurrence rate.

It is of great importance to determine the timing of wide surgical excision and the selected method of reconstruction. During the acute phase, surgical drainage, irrigation with the administration of antibiotics should only be the mainstay of the treatment. Our approach has not been conducted in this phase. It was important to obtain a non-infectious wound to perform this approach and not to expect septic complications. Then, planning of reconstruction should be initiated to achieve the best outcome and consequently decreasing the risk of recurrence and complications after surgery.

## Conclusion

Treatment of hidradenitis suppurativa has wide modalities. However, surgical option can be the option of treatment especially for those severe cases being treated with conservative modalities. After eliminating the acute inflammatory phase, wide surgical excision is recommended and planning of reconstruction should be initiated to achieve the best outcome and consequently decreasing the risk of recurrence and complications after surgery. Our concept of treatment including reconstructive techniques decreased the time of hospital stay. It is cost effective and prevented skin contractures as well as excessive scarring and showed good functional and aesthetic results.

## Competing interest

The authors have declared that no conflict of interest exists.

## Authors’ contribution

ZA drafted the manuscript and participated in the analysis and interpretation of data. JK conceived the design and participated in the operations. NP participated in the operations as well as revised and edited the manuscript. All authors read and approved the final manuscript.

## Pre-publication history

The pre-publication history for this paper can be accessed here:

http://www.biomedcentral.com/1471-5945/12/9/prepub
